# Inhibitors of Discoidin Domain Receptor (DDR) Kinases for Cancer and Inflammation

**DOI:** 10.3390/biom11111671

**Published:** 2021-11-10

**Authors:** William A. Denny, Jack U. Flanagan

**Affiliations:** 1 Auckland Cancer Society Research Centre, Maurice Wilkins Centre, School of Medical Sciences, University of Auckland, Auckland 1142, New Zealand; j.flanagan@auckland.ac.nz; 2 Department of Pharmacology and Clinical Pharmacology, School of Medical Sciences, University of Auckland, Auckland 1142, New Zealand

**Keywords:** small molecules, selectivity, DDR kinase inhibitors

## Abstract

The discoidin domain receptor tyrosine kinases DDR1 and DDR2 are distinguished from other kinase enzymes by their extracellular domains, which interact with collagen rather than with peptidic growth factors, before initiating signaling via tyrosine phosphorylation. They share significant sequence and structural homology with both the c-Kit and Bcr-Abl kinases, and so many inhibitors of those kinases are also effective. Nevertheless, there has been an extensive research effort to develop potent and specific DDR inhibitors. A key interaction for many of these compounds is H-bonding to Met-704 in a hydrophobic pocket of the DDR enzyme. The most widespread use of DDR inhibitors has been for cancer therapy, but they have also shown effectiveness in animal models of inflammatory conditions such as Alzheimer’s and Parkinson’s diseases, and in chronic renal failure and glomerulonephritis.

## 1. Introduction

**Figure 1:**
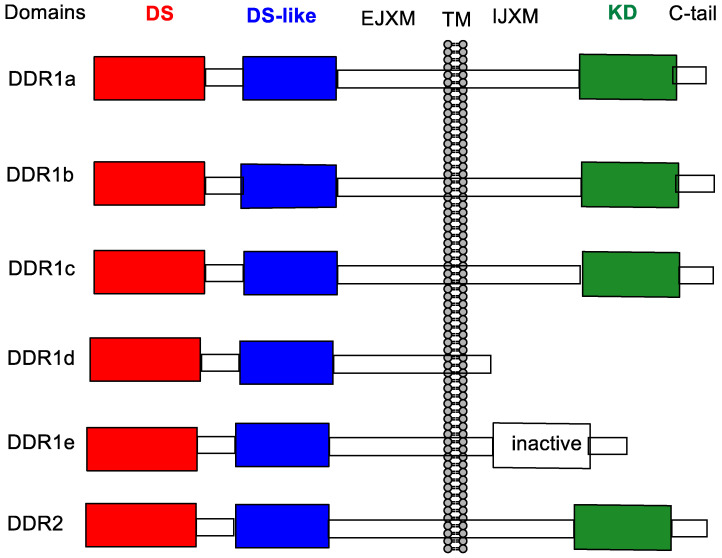
Generic structures of DDR1 and DDR2. DS, N-terminal discoidin domain; DS-like, discoidin-like domain [3]; EJXM, extracellular juxtamembrane domain; TM, trans-membrane domain; IJXM, intracellular juxtamembrane domain; KD, kinase domain. After [10].

The discoidin domain kinase inhibitors DDR1 and DDR2 conform to the broad structural organisation of receptor tyrosine kinase enzymes (cellular domain, membrane domain, extracellular domain), but are distinguished from the other 58-odd human receptor tyrosine kinases in that their extracellular domains interact with collagen rather than with peptide-like growth factors [1]. Collagen is comprised of three left-handed amino-acid helices that twist to form a right-handed triple helix, with further association of these to form more complex microfibrils. DDR1 comes in five isoforms (DDR1a–1e) while DDR2 has only one isoform (Figure 1). The expression profiles in cells are also quite different; the DDR1 variants are widely expressed in many tissue types, with expression of DDR2 being limited to mesenchymal type cells [2].

Unlike most growth-factor-receptor-linked tyrosine kinases, which are activated by dimerization upon ligand binding and autophosphorylation, DDRs have to dimerize to allow binding (to collagen) and then initiate tyrosine phosphorylation [3,4]. Many papers have shown that the over-expression/mutation of DDR1 is associated with multiple cancers, including major types such as lung, breast, brain, liver, pancreas and prostate [5], and that this over-expression/mutation promotes significant disease progression on a large number of non-cancer disease states caused by various inflammatory conditions, including fibrosis [6], atherosclerosis [7] and liver/kidney dysfunction [8,9].

## 2. Approved Cancer Drugs with Significant DDR1/2 Activity

To date, the most important use of DDR inhibitors has been in cancer therapy, and there have been several informative reviews on this aspect of their development and use [5,10]. It was shown quite early on that, because the kinase domains of DDR1 and DDR2 share quite high sequence and structural homology with those of both the c-Kit and Bcr-Abl kinases [5], many of the DDR1/2 inhibitors are also effective inhibitors of these kinases (Table 1; compounds **1**–**7**). 

**Figure 2:**
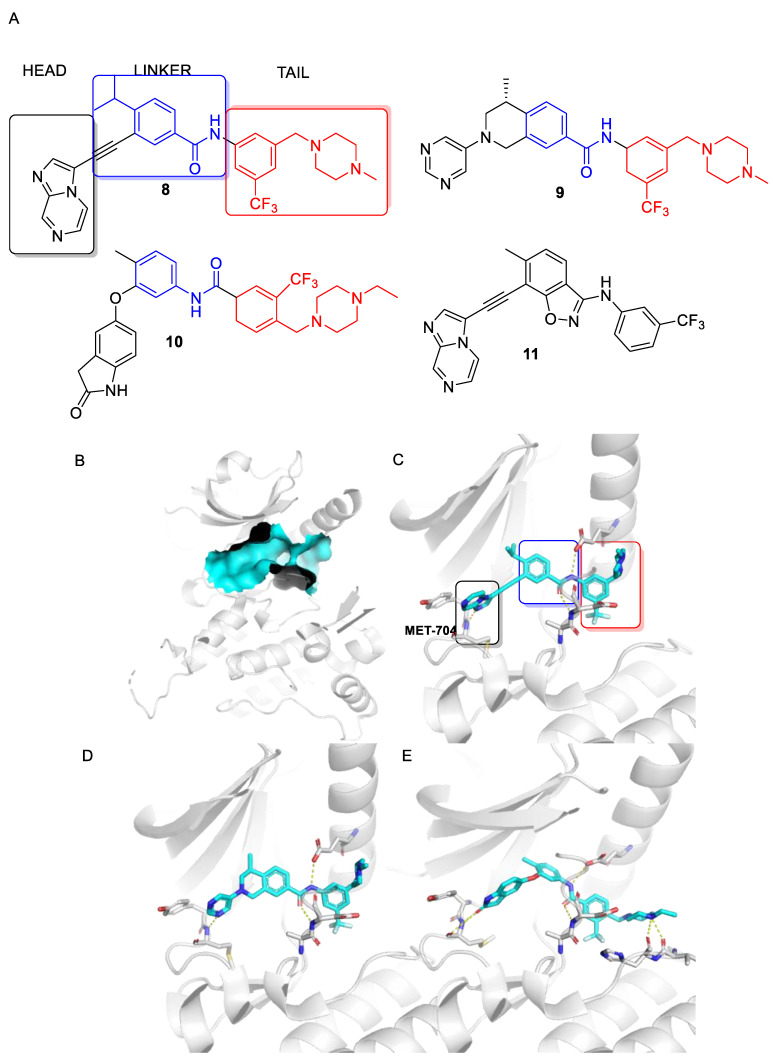
(**A**) DDR inhibitors can be divided into three regions based on the location in the ATP binding site. (**B**) ATP binding site location between the N- and C-terminal lobes of the kinase domain (cyan surface). (**C**–**E**) Binding modes for compounds **8**–**10** in the DDR1 ATP binding site (PBD codes 6GWR, 5FDP and 4CKR, respectively). The colored boxes represent the head (black), linker (blue) and tail (red) units. The hydrogen bond with Met-704 is indicated with the dashed bond. Hydrogen bonds with the side chain of Glu-672 and with the backbone amide of Asp-784 are also shown. (Images created with PyMol).

These, and many other such multi-kinase inhibitors have a broadly similar structure; overall quite linear molecules bearing a potential H-bond acceptor on an aromatic/polyaromatic unit on one end and usually a “solubilizing” unit on the other, linked via a small series of “rotatable” aromatic units. Several studies [13,14,15,16] with the respective reasonably disparate compounds **8**–**11** show their varying terminal aromatic units (Figure 2A).

Most DDR inhibitors bind to the kinase domain and can been considered as three distinct units that transition from the ATP binding site where most inhibitors form a hydrogen bond to Met-704, into a hydrophobic selectivity pocket and linker region created by rotation of the DFG motif and movement of the αC helix. 

## 3. The DDR Kinase Domain 

X-ray crystal structures of the kinase domain for both DDR1 and DDR2 have been determined. These illustrate that the DDR kinase domain has a structure common to other protein kinases with the ATP binding site located in a cleft between two lobes. The structures include an auto-inhibited state that illustrates how the ATP binding site is blocked by the activation loop in the inactive state of the enzyme, and inhibition is released first by phosphorylation of Tyr-569, located on a segment outside of the kinase region [17]. The majority of inhibitor-bound structures available in the Protein Data Bank are for DDR1, with only a few available for DDR2. In general, the kinase ATP binding site can adopt multiple conformations with the regulatory elements including the DFG tripeptide and the αC helix used to classify inhibitors. ATP competitive inhibitors are classified as Type I and Type II based on the conformation of the DFG motif. Type I inhibitors bind to an active conformation, and Type II bind in an inactive conformation [18]. All DDR ATP competitive inhibitors to date can be classified as Type II inhibitors binding to a DFG-out ATP site conformation. In these structures, the αC helix has variable positions, and according to the automated annotation within the KLIFS database [19] for most available structures these can be classified as occupying the “in” position considered necessary for activity. Most DDR inhibitors form a hydrogen bond with the backbone amide of Met-704 in the linker region (Figure 2C–E). As they transition from the ATP binding site, through the hydrophobic selectivity pocket, other hydrogen bonds can also be made between the linker region and the backbone amide of the centrally located Asp-784 found in the DFG motif and the sidechain carboxyl of the αC-helix Glu-672 as illustrated in Figure 2C–E for compounds **8**–**10** before accessing a hydrophobic back pocket with the tail.

## 4. Selective DDR1 Inhibitors

**Figure 3:**
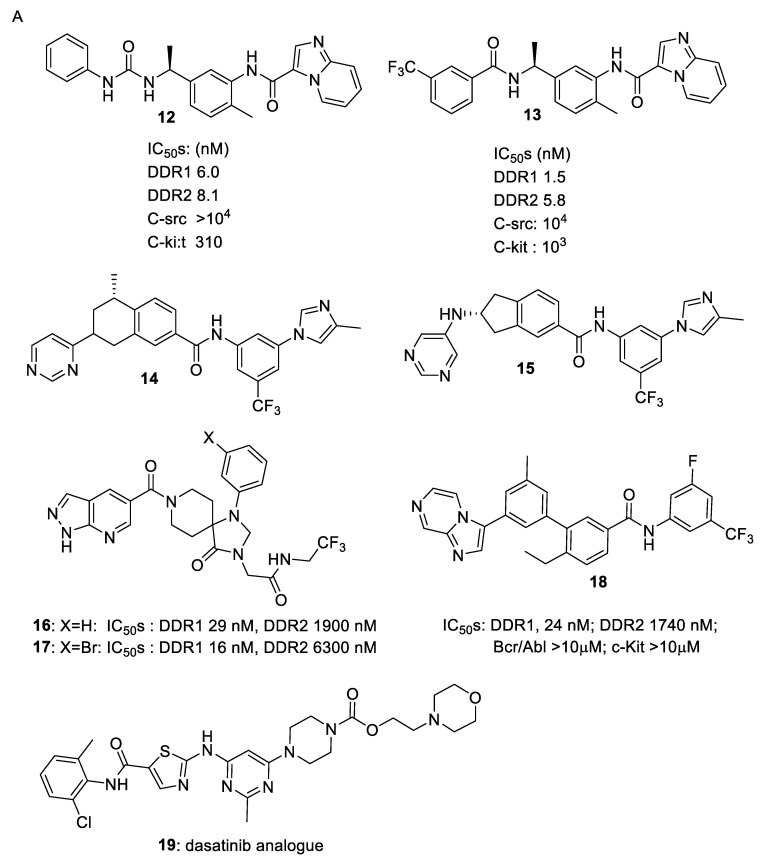

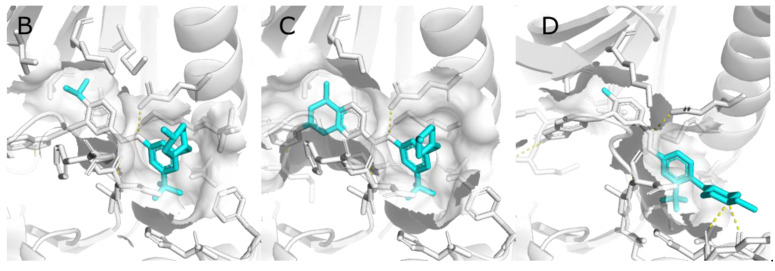
(**A**) Selective DDR inhibitors. (**B**–**D**) Hydrophobic back pocket and selectivity pockets targeted by some selective compounds **8**–**10** (PBD entries 6GRW, 5FDP and 4CKR, respectively) (image created with PyMol).

As noted above (Table 1), many DDR1/2 inhibitors are also effective Bcr-Abl/c-Kit inhibitors, due to the structural similarity of the respective ATP binding pockets of these enzymes. However, Murray et al. [20] noted differences in the ATP–phosphate binding loop (P-loop) of these enzymes that could be exploited to provide more DDR1/DDR2-selective compounds (e.g., **12**, **13**). They used a chemotype fusion strategy guided by the superposition of a fragment hit that binds to a DFG-out αC-in conformation motif and the DDR1 dasatinib structure which also selects the same ATP site conformation (Figure 3B). 

Wang et al. [21] evolved, from an earlier lead (**10**; DDR-IN-1) the much more potent DDR1 inhibitor **14** and showed that it was highly selective for DDR1 (IC_50_ 9.4 nM) in a 468-kinase panel. Analysis of inhibitor binding to the Abl ATP binding site illustrated important π–π interactions with Tyr-253 that were unnecessary for binding to the DDR1 ATP site. Inhibitor design set out to remove these π–π interactions and avoid additional π–π interactions with Abl Phe-382. Molecular docking into DDR1 and the off-target Abl kinase indicated that the initial designs could fit DDR1 but not the Abl ATP binding sites. It was also active in an in vivo mouse model of bleomycin-induced lung fibrosis. Gao et al. [22] showed that the hydrophobic back-pocket could be used to achieve selectivity over Abl kinase, and demonstrated that substitutions at this site as well as those at the flag-methyl accessing the hydrophobic selectivity pocket had a coordinated effect. Kim et al. [15] also demonstrated the utility of the hydrophobic back pocket (Figure 3A–C). Zhu et al. [23] developed a series of indene-5-carboxamides (e.g., **15**; DDR1 IC_50_ 5.6 nM). Compound design was guided by the comparison of predicted binding modes in DDR1 to those in an off-target kinase TrkC followed by the comparison between DDR1 and a homology model of DDR2, and the compounds were effective against in vivo orthotopic mouse models of pancreatic cancer. Richter et al. [24] used DNA-encoded library screens against DDR1 and DDR2 to discover a novel series of spiro inhibitors with good selectivity for DDR1 over DDR2 as well as broad selectivity against the wider cellular kinome. Co-crystal structures of compounds were obtained and used to guide the design of new analogues. Examples such as **16** and **17** selectively inhibit DDR1 over DDR2 phosphorylation in vitro and prevent collagen-induced activation of renal epithelial cells expressing DDR1. Compound **17** showed better metabolic stability, provided improved renal function, and reduced fibrosis and tissue damage in a preclinical mouse model of Alport syndrome (progressive loss of kidney function) and in a model for chronic kidney disease. Mo et al. [25] interrogated a predicted binding model identifying chemical features that could control potency and selectivity and identified a series of highly DDR1-specific biphenylcarboxamides, highlighting compound **18**, which suppressed NSCLC cell tumorigenicity, migration and invasion.

Since human melanoma specimens show a correlation between high DDR1 expression in melanoma lesions and poor disease prognosis, de Moura et al. [26] suggest that DDR1-selective inhibitors may be of therapeutic value in this disease. They showed that the DDR1 inhibitor **10** (DDR1-IN-1; DDR1 enzyme IC_50_ 105 nM) inhibited melanoma cell proliferation in both in vitro and ex vivo assays of hereditary Alport syndrome, tubular obstructive nephropathy and nephrotoxic serum glomerulonephritis, and was effective in animal melanoma xenograft models. Liu et al. [27] reported the design and evaluation of novel dasatinib analogues as DDR1 and DDR2 inhibitors. Compound **19** was superior to dasatinib (**7**) against both DDRs, and demonstrated potent inhibitory activity against K562 cell lines (IC_50_ values of 2.26 nM for DDR1, 7.04 nM for DDR2 and 0.125 nM for the K562 cell line).

**Figure 4:**
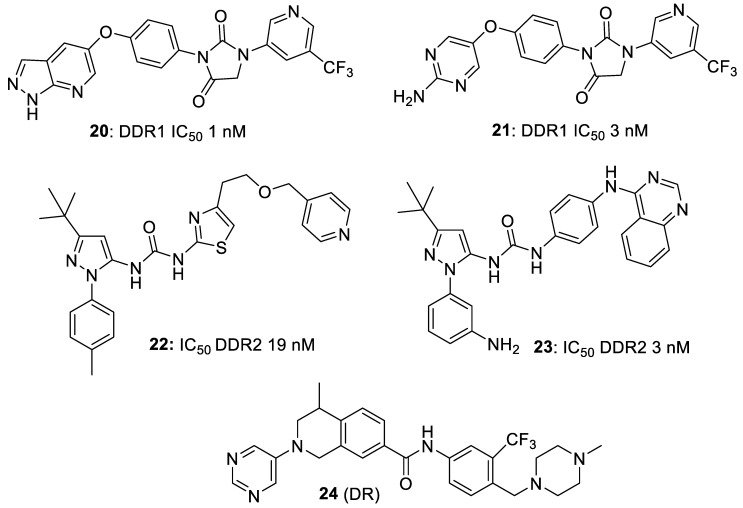
Selective DDR1 inhibitors.

Nishiota et al. [28] reported a series of hydantoin-type compounds (e.g., **20** and **21**) that were very potent inhibitors of DDR1 (IC_50_s 1 and 3 nM, respectively), with suggested utility in cancer, kidney disease, or fibrosis. Using a fluorescence assay, Richters et al. [29] were able to distinguish between type II and III DFG-out binders for DDR2 and generated a series of potent DDR1/2 inhibitors (e.g., **22** and **23**), with DDR2 IC_50_s of 19 and 3 nM, respectively. Xu et al. [30] used a quantitative proteomics approach to identify the major target of the tetrahydroisoquinoline-based compound **24** (DR) as a selective DDR1 inhibitor (IC_50_ 21 nM) with cathepsin D as a major off-target protein in cancer cells (Figure 4). 

## 5. Allosteric Inhibitors

**Figure 5:**
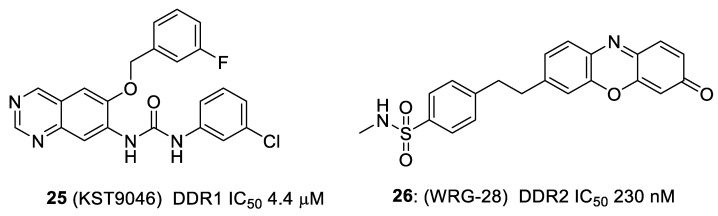
Allosteric DDR inhibitors.

As noted above, Sammon et al. [17] noted that the reported two-step release from autoinhibition by the kinase-proximal segment (JM4) of DDR1 could be useful in the design of allosteric, DDR1-specific kinase inhibitors. Elkamhawy et al. [31] proposed that their quinazoline-urea compound **25** (KST9046) was an allosteric inhibitor of DDR1 acting through the ATP binding site on the basis of their molecular docking studies, while Grither and Longmore [32] developed the DDR2 allosteric inhibitor **26** (WRG-278) (Figure 5). This compound is an allosteric inhibitor that binds to the extracellular region between the discoidin and discoidin-like domains.

## 6. Other Types of DDR Inhibitors in Patent Claims

**Figure 6:**
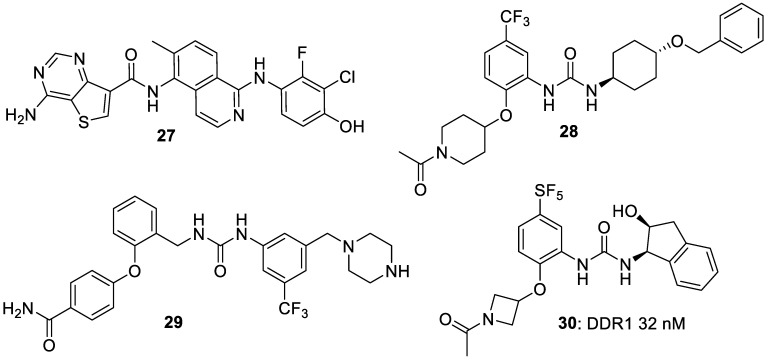
DDR inhibitors in the patent literature.

Bae et al. [33] claimed thieno[3,2-d]pyrimidines (e.g., **27**) as inhibitors of kinases including the DDR kinases, for potential use in disease caused by over-expression of RAS protein or its associated kinases. Nishio et al. [34,35] reported studies of phenylureas **28** and **29** as DDR kinase inhibitors with potential uses in cancer treatment; **28** had an IC_50_ of 4.2 nM for inhibition of DDR1. Tsutsui et al. [36] employed the relatively unusual SF_5_ group in a series of chiral ureas as DDR1 inhibitors (e.g., compound **30**), which had a DDR1 IC_50_ of 32 nM (Figure 6).

A recent review [10] of patent claims in the area covers other examples.

## 7. DDR1 Inhibitors in Cancer Treatment

The most widespread use of DDR inhibitors has been for cancer therapy. Dong et al. [37] developed a series of pyrazolo[3,4-d]pyrimidine derivatives, of which **31** blocked the proliferation of the DDR1-overexpressing cell lines HCT-116 and MDA-MB-231 (IC_50_ values of 4.0 and 3.4 μM, respectively). Kim et al. [15] reported the activity of **10** (DDR1-IN-1; Figure 2A) in a variety of cancer cell lines. The compound has a rather unusual benzofuranone H-bond acceptor that targeted the Met-704 residue in the hydrophobic pocket of the enzyme. It is moderately selective for DDR1 over DDR2 (IC_50_s 105 and 431 nM, respectively).

Compound **10** has been widely explored and shown to be effective against a large range of different cancer cell types. Vehlow et al. [38] showed that the expression of DDR1 in glioblastoma correlated with poor clinical outcome, and that inhibition of DDR1 with **10** together with radiotherapy gave better survival than conventional therapy in animal models of GBM. Romayer et al. [39] showed that colon carcinoma secretomes increased DDR1 phosphorylation in hepatic stellate cells, and that inhibition of DDR1 with **10** decreased the expression of chemoattractant and proliferative factors and reduced tumor metastasis in animal models, while De Moura et al. [26] showed that down regulation of DDR1 with **10** reduced migration, invasion and survival in several human melanoma cell lines.

**Figure 7:**
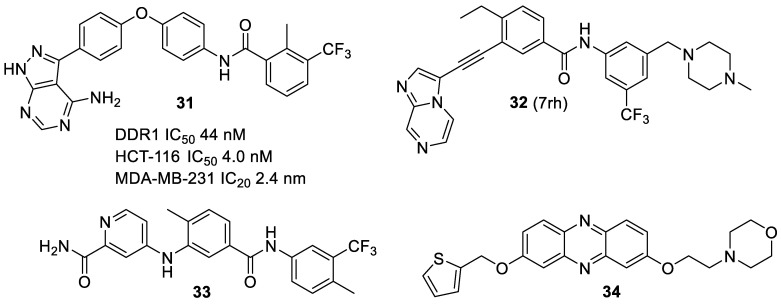
DDR inhibitors in cancer treatment.

Gao et al. [22] reported the synthesis and characterization of a close analogue of **7**, the ethylated compound **32** (7rh). This was shown to be a selective inhibitor of DDR1, with an IC_50_ of 6.8 nM, but was much less effective against DDR2, Brc-Abl and c-Kit, and showed good bioavailability. It has been extensively explored in biological assays. In a study of highly drug-resistant triple-negative breast cancer MDA-MB-231 cells with high levels of type I collagen (which favors metastasis and drug resistance) the best sensitization was shown with combinations of **32** together with ERK1/2 or EGFR inhibitors [40]. Hur et al. [41] showed that 50% of human gastric carcinomas were positive for expression of DDR1, and that this constituted a poor prognosis, but that treatment with **32** suppressed tumor growth in gastric cancer xenograft models. In pancreatic cancers, Aguilera et al. [42] showed collagen-induced activation of DDR1 by pro-tumorigenic signaling through the kinases PYK2 and PEAK1, and that inhibition of DDR1 with **32** showed high efficacy, in combination with chemotherapy, in orthotopic xenografts of pancreatic adenocarcinomas. Lu et al. [43] explored the effects of **32** on the nasopharyngeal carcinoma cell lines CNE2 and HKI, found IC_50_s of 2.60 and 6.33 μmol/L, respectively, and suggested that implied that dual inhibition of DDR1 and IGF-1R may be a suitable therapeutic approach (Figure 7).

Borza et al. [44] show that blocking DDR1 kinase activity with the ATP-competitive small molecule inhibitor **33** reduces collagen production, indicating that the kinase activity of DDR1 plays a key role in DDR1-induced collagen synthesis and suggest that blocking collagen-mediated DDR1 activation may be beneficial in fibrotic diseases. Jin et al. [45] explored the use of **34** in gastric carcinoma, since screening of gastric cancer patients showed that 50% had enhanced DDR1 expression, which correlated with poorer prognosis. In athymic nude mice it suppressed the growth of gastric cancer xenografts and reduced phosphorylated DDR1.

In a recent patent application, Rovati et al. [46] claimed alkoxyphenazine analogues (e.g., **34**), which inhibited the production of the DDR2 receptor in HEK293 cells with an IC_50_ of 1.1 μM, as useful in the treatment of (DDR2)-mediated diseases such as cancer and inflammation.

Vehlow et al. [47] showed that DDR1 expression causes glioblastoma resistance to therapy through adhesion to extracellular matrix and modulating autophagy, and that inhibition of DDR1 re-sensitizes cells to therapy by inducing autophagy.

Yuge et al. [48] noted that while DDR1 expression in human gastric tumors is associated with poor prognosis, MKN74 gastric cancer cells which were DDR1-silenced still showed proliferation activity but migration, invasion and tube formation were significantly reduced in comparison with the wild-type cells. They suggested that DDR1 inhibits multiple steps of the gastric cancer metastasis process. Zhong et al. [49] showed raised levels of DDR1 expression in human breast tumors compared with adjacent normal tissues, and that over-expression of DDR1 in murine breast cancer 4T1 cells promoted tumor growth in mouse models that could be decreased with DDR1-neutralizing antibodies.

## 8. DDR Inhibitors for Inflammatory Conditions

**Figure 8:**
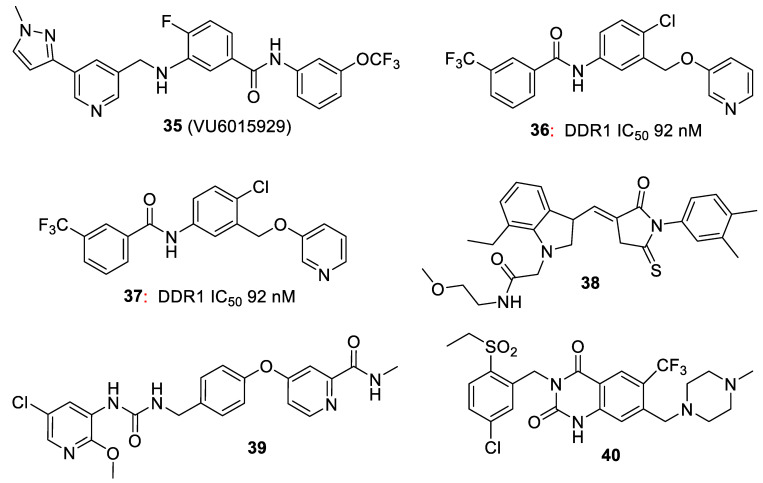
DDR inhibitors for inflammatory conditions.

In addition to their growing usefulness in cancer therapy, DDR1/2 inhibitors are also useful in the treatment of various inflammatory conditions. Wang et al. [16] showed that the R enantiomer (**9**) of the tetrahydroisoquinoline class of DDR inhibitors showed good oral activity in a bleomycin-induced mouse model of pulmonary fibrosis. Jeffries et al. [13] optimized a series of pan-DDR1/2 inhibitors to give the more potent **35** (VU6015929), which was shown to block collagen-I-induced DDR1 autophosphorylation in cells much more effectively, with IC_50_s of 4.7 and 0.71 nM, respectively (Figure 8), suggesting its use in antifibrotic therapy (Figure 8).

Yoshimori et al. [50] used a machine learning “deep generative model” process (Atsushi et al. [50,51,52]) to automate the design of DDR1 inhibitors for fibrosis and cancer. The most potent of these was the benzamide **36**, which had an IC_50_ of 92 nM for inhibition of DDR1.

Dorison et al. [6] discussed the role of DDR1 in renal physiopathology, including hypertensive nephropathy, tubular obstructive nephropathy and nephrotoxic serum glomerulonephritis, and the use of DDR1 inhibitors in the treatment of such diseases. Lino et al. [52] showed that DDR1−/− mice on a high-fat diet had improved glucose tolerance, reduced body fat and increased brown fat activity and energy expenditure compared to DDR1+/+ littermate controls, correlating with adipose tissue expansion and fibrosis mice on a high-fat diet, indicating the potential value of DDR inhibition for these conditions. Ushakumary et al. [53] used male rhesus monkeys as a non-human primate model to show that a high-fat, high-sucrose diet induced a marked increase in DDR2 and collagen type I levels, enhanced fat deposition and calcification in the abdominal aorta, and arterial fibrosis, supporting DDR2 inhibition as a therapeutic target. Mattison et al. [54] had previously shown similar results.

Sun et al. [55] showed that the thienopyridine analogue LCD-03-0110 (**37**) is a potent inhibitor of the DDR and other c-SRC family kinases and suppressed scar formation by inhibiting fibroblast and macrophage activation. Zhao et al. [56] noted that idiopathic pulmonary fibrosis (IPF) is a lethal human disease with limited treatment options, but showed in mouse studies that even late-stage treatment with either a specific DDR2 siRNA against DDR2 or dasatinib (**7**); a potent DDR2 inhibitor (IC_50_ 5.4 nM) [56] were effective. Terai et al [57] showed that inhibition of both DDR2 and SRC led to enhanced suppression of DDR2 mutant lung cancer cell lines.

In a search for inhibitors of DDR1 for inhibiting fibrosis, Chen et al. [58] carried out a screen based on enzyme structure, molecular dynamics and a docking-based virtual screening method, which led to **38** as the most potent compound. However, this 5-thioxopyrrolidin-3-ylidene is a classic PAIN (pan assay interference compound) [59], and thus unlikely to proceed further. Chou et al. [60] studied the effect of **38** in osteoarthritis in 8-week-old mice and showed that treatment with the compound decreased cartilage degradation and significantly improved performance (weight-bearing and running). They suggested that it represents a potential disease-modifying therapy to slow the progression of osteoarthritis.

Wucherer-Pleitker et al. [61] claimed a large number of pyridinylpicolinamide DDR1/DDR2 inhibitors (e.g., **39** (IC_50_s DDR1 37 nM, DDR2 6.8 nM) showed promise for the treatment of osteoarthritis and other inflammatory diseases.

Murata et al. [62,63] described the preparation of a series of related quinazolinedione derivatives (e.g., **40**; DDR1 IC_50_ 21 nM; DDR2 IC_50_ 450 nM) as DDR inhibitors with potential use in inflammation.

## 9. DDR Inhibitors in Brain Disease

**Figure 9:**
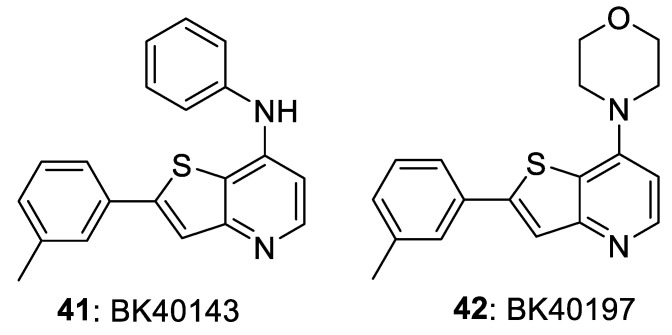
DDR inhibitors in brain disease.

Fowler et al. [64] evaluated kinase inhibitors in transgenic mouse models of Alzheimer’s and Parkinson’s diseases and showed that combinations of nilotinib (**2**) (multi-kinase Abl/DDR inhibitor), LCB-03-0110 (**37**) (DDR/Src inhibitor) and bosutinib (**7**) (Abl/Src inhibitor) were superior to the more selective Abl inhibitors radotinib (**6**) and bafetinib (**4**) (Figure 9).

Fowler et al. [12] also prepared the thieno[3,2-b]pyridinamines **41** (BK40143) and **42** (BK40197) and evaluated them in a mouse model that recapitulated vascular dementia (age-related accumulation of Aβ and *p*-tau proteins and neuroinflammation). Both compounds lowered the levels of DDR1 and neurotoxic proteins, and increased autophagy and inflammation in the brains of mice, demonstrating that DDR1 is a therapeutic target for neurodegeneration.

## 10. DDR Inhibitors in Renal Disease

Chronic renal failure—a common complication of hypertension—is associated with the abnormal accumulation of collagens in renal tissue. Flamant et al. [65] demonstrated the influence of DDR1 in this process by showing that renal fibrosis and inflammation were significantly reduced in hypertension-induced DDR1-deficient mice compared with normal animals, supporting the value of DDR1 inhibitors in this disease. Kerroch et al. [66] similarly showed that DDR1-deficient mice were protected from induced glomerulonephritis, and that wild-type animals were similarly protected by the administration of DDR1-specific antisense oligodeoxynucleotides.

## 11. Conclusions

The work summarized in this review highlights the remarkably rapid development of DDR inhibitors. This was initially sparked by the overlapping activity of previously developed c-Kit and Bcr-Abl inhibitors, resulting from the relatively high sequence and structural homology of their kinase domains with those of the DDRs. Since then, guided by a good understanding of the molecular-level 3D structures from DDR/drug crystallography, potent and highly selective compounds have been developed (even between DDR1 and DDR2 isoforms; e.g., compound **17**: DDR1/DDR2 enzyme IC_50_s 16 and 6300 nM, respectively). The application of these inhibitors to an increasing range of serious inflammatory conditions (fibrosis, renal, arthritis, diabetes etc.) bodes well for the continued development of the discoidin domain kinase inhibitors.

## Figures and Tables

**Table 1:** DDR inhibition profiles of some approved DDR-binding drugs.

Name	Structure	IC_50_ (nM)			
		DDR1	DDR2	Bcr-Abl	c-Kit
**1**: Imatinib [11]	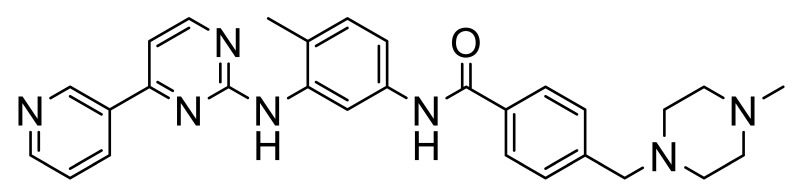	337	675	25	5.4
**2**: Nilotinib [11]	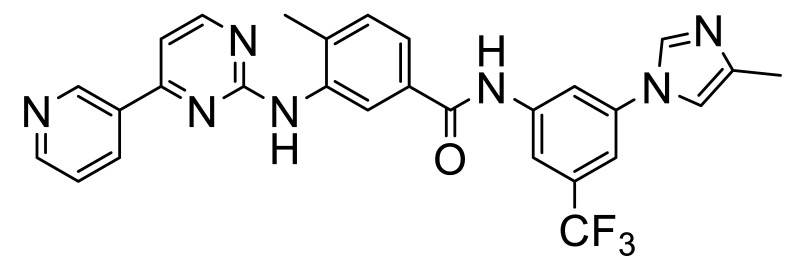	43	55	25	26
**3**: Ponatinib [11]	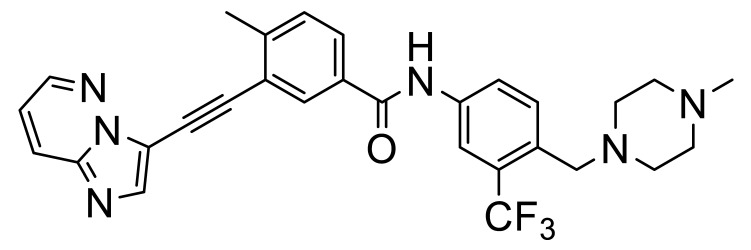	9	9	2	12
**4**: Bafetinib [12]	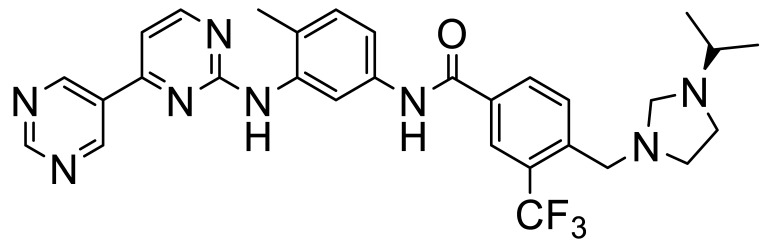	220		5.2	
**5**: Olverembatinib	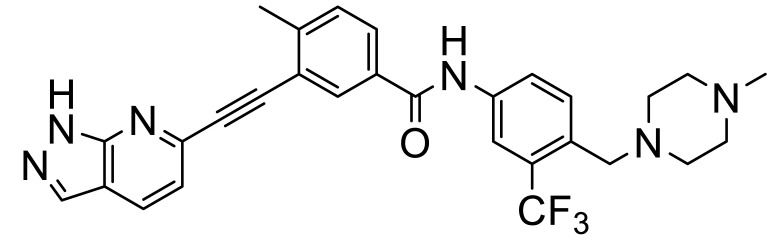			1.0	
**6**: Radotinib [12]	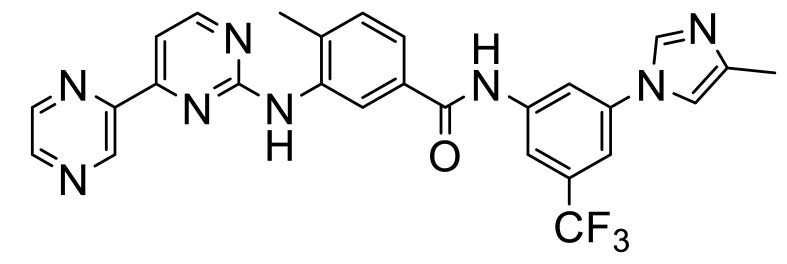	180		34	
**7**: Dasatinib [11]	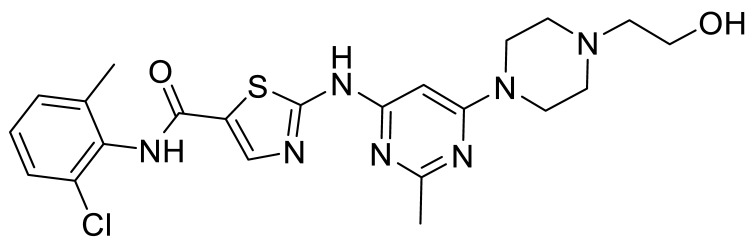	0.5	1.4		30

## Data Availability

All data reported are contained in the listed references.
